# Overcoming the monster! Perceptions of physiotherapy students regarding the use of stroke master plots for building therapeutic relationships; a vignette study

**DOI:** 10.1186/s12909-023-04301-5

**Published:** 2023-05-05

**Authors:** Rana Alawafi, Sheeba Rosewilliam, Andrew Soundy

**Affiliations:** 1 School of Sport, Exercise and Rehabilitation Sciences, University of Birmingham, Birmingham, B15 2TT UK; 2 King Khalid International Airport, And Princess Nourah Bint Abdulrahman University, Riyadh, 13412 Saudi Arabia

**Keywords:** Stroke, Neurology, Physiotherapy students, Illness narratives, Master plot

## Abstract

**Background:**

Narrative master plots identify illness stories which are recognisable within clinical settings. Responses to different master plots by physiotherapy students can lack empathy and need to be understood further. One narrative master plot for people with stroke that has not been well studied is called ‘overcoming the monster’. Research is needed to understand physiotherapy students’ reactions to this master plot.

**Objective:**

To examine the responses of physiotherapy students to three variations of the master plot called ‘overcoming the monster’ generated from patients who have had a stroke.

**Methods:**

A qualitative narrative vignette study was undertaken. A university in the West Midlands (England) was used to access physiotherapy students on the pre-registration programs. A purposive sample of students volunteered to complete a single vignette questionnaire at one time point. The vignette provided three unique examples of the master plot overcoming the monster as told by people with stroke. Students responded to each by asking specific questions that captured demographic information and questions that captured reactions to the different versions of the master plot. Categorical-content narrative analysis was undertaken.

**Results:**

Thirty-two first year (BSc) students, thirty-nine first year (pre-registration) MSc students and nineteen third year (BSc) students participated in this study. Neither first year groups had undertaken any clinical placement hours. All third-year students had finished the required clinical placement hours for the physiotherapy course. Students consistently demonstrated empathy towards this master plot. Students often valued the variant of the story which illustrated how difficulties following stroke could be experienced as an ‘adventure’. Students also valued and were motivated by the story variant that considered a family member as a source of motivation and encouragement. The story variant which focused on the shortcomings of the health care system was more often related to by the final year BSc students and MSc students. However, first year students, particularly BSc students, reported being more emotionally affected by the vignette.

**Conclusion:**

All variants of the master plot overcoming the monster appeared to generate empathetic responses. This is important because it highlights the value of students’ understanding the patients’ story and challenges or ‘monsters’ faced. Therapeutic relationships will benefit from training physiotherapy students on the importance of listening and exploring challenges people with stroke face.

**Supplementary Information:**

The online version contains supplementary material available at 10.1186/s12909-023-04301-5.

## Background

According to the Stroke Association [1] a stroke is defined as an event which affects the blood supply to the brain, which kills brain cells and affects how the body works. Stroke is the world’s second leading cause of mortality and the leading single cause of disability [2]. Stroke is a devastating condition with complex long-term physical, cognitive, psychological, and functional disabilities that demand care and support [3]. Student health care professionals are required to deliver a patient-centered approach to care [4]. Fundamental to this approach is the therapeutic relationship between patients and (student) health care professionals [5]. Therapeutic relationships can positively influence patient outcomes during stroke rehabilitation, as well as satisfaction of services received [6, 7]. Communication is central to establishing an optimal therapeutic relationship. Poor communication can have a negative impact on patients’ psychosocial well-being [8]. There are specific risks to the quality of communication between patients and healthcare professionals such as stress from heavy caseloads [5] or inherent biases toward the patients [9]. There is a need to pay attention to the teaching skills provided to student healthcare professionals that help develop better communications skills and hence improved therapeutic relationships for people with stroke [10, 11].

Sharing illness narratives is one approach that can build therapeutic relationships between patients with stroke and (student) healthcare professionals [12]. Research [13] with physiotherapists and people who have suffered a stroke identified several benefits of sharing stories, this includes patient empowerment and greater involvement and shared decision making. The opportunity to share a personal narrative following stroke aids the ability to make meaning from the experiences and recover emotionally and socially [14]. Hence, storytelling interventions can aid the psychological health and emotions of patients who have had a stroke [15]. Healthcare students like physiotherapists need to be aware of these benefits.

Illness narrative master plots are recognisable stories of illness identifiable by the overarching plot often told in relationship to a particular and unique experience of illness. Research [16] has identified 13 master plots told by people with neurological illnesses. For instance, these include plots like the detached master plot which views events of the illness in a detached way with little emotion, the comic master plot which views the experiences of illness differently, or the detective master plot which illustrates a search for answers following illness. Recently, research has extended this understanding to more master plots. One significant master plot is called ‘overcoming the monster’. This narrative master plot was defined by Booker [17] as a major plot existing in literature. The plot relates to stroke as the different experiences related to, and following the onset of stroke can represent a monster which needs to be overcome. Examples of a ‘monster’ for people following a stroke include interpersonal challenges, mental health challenges, isolation and changes to their social identities [8]. Physiotherapy students need to be aware of the importance of this master plot and understand how stories can be empowered or disempowered through interactions [18].

There is a paucity of research considering physiotherapy students’ reactions to different illness narrative master plots or the impact of this reaction on stroke care provision [19]. One way to understand physiotherapy students’ reaction to stories is to use a vignette. A vignette is a demonstrative account of specific points in a story, based on the voices and perspectives of various participants [20]. Vignette based research on physiotherapy students has highlighted that empathy can be lost through training and that students in later years with clinical experience can focus their responses on what is wrong with the plot [19]. Overcoming the master plot is different from past master plots because it does not challenge the student’s understanding of what may be possible in rehabilitation rather it reveals what is possible and has been achieved. Research is needed that identifies how student healthcare professionals can work more effectively with different narrative master plots [8, 15] and using different stories that show how people change post stroke [21].

Given the above, the aim of this study was to gain an insight into how physiotherapy students perceive and react to three vignettes that represent the master plot called overcoming the monster from people following a stroke.

## Method

### Design

A narrative vignette [19] was used. In this case, it included providing three narrative master plots identified as overcoming the monster each with a different ‘monster’ identified from individuals who had suffered a stroke.

### Setting

A university in the west midlands was selected for the research during the academic year of 2021/2022. The vignette was given to student physiotherapists in written formats during a lecture or via an email for an online form to be completed.

### Sampling and sample size

A purposive sample was taken from two cohorts of undergraduate pre-registration physiotherapy students first year (BSc) and final year (BSc) students and one cohort of pre-registration master (MSc) physiotherapy students. First year BSc and MSc students had never been taught about illness narratives. Third-year students, on the other hand, were familiar with concept of narratives and listening and understanding stories.

We considered sample size based on the concept of information power [22]. The principles of information power are that authors should consider the aim of the study, the sample used, the type of analysis, identification of any established theory and quality of the data. A previous study [19] used 122 (77 BSc Year 1 and 45 BSc year 3) students in total and focused on three different narrative master plots using very similar questions for analysis. Given the greater focus on one narrative master plot it was identified that a similar number would be appropriate. Given the past study had similar aims, questions asked and analysis strategy we believed using the same strategy of requesting response from three cohorts of students would provide a useful number of responses to contrast between the three groups.

### Procedure

Physiotherapy students were contacted on a single occasion by A.S via a generic email to each year group email address. The study was also presented at the start of a nominated module where all students were present. Physiotherapy students were not being taught by A.S at that time point of the study. Although students of all cohorts would know him. If students responded by email, the follow-up email provided the ethically approved information about the study and identified that if the student was happy to undertake the study. Students were required to click on a link for the vignette. Alternatively, students could have received it as a hard copy in a lecture. If students received a hard copy of the vignette it was given out by a separate module leader on one identified occasion to any students who wanted to complete it. The researchers were not in attendance to see who did or did not complete the vignette but collected the completed sheets from the session’s lecturer after the lecture was over. The vignette was accompanied by a survey using a questionnaire.

The qualitative vignette itself was created by R.A and A.S. The development of the vignette questionnaire was based on past research [19]. The reason for this was because the previous questionnaire study provided useful information about students’ perceptions and reactions to a narrative. Moreover, this also provided a way to compare responses of physiotherapy students to past responses.

### Outcome measure

Demographic data was identified within the vignette questionnaire. The questions were designed to elicit students’ reactions and perception about the vignette. The vignettes were created within a three-step process based on work conducted prior to this vignette study. Step 1 included authors reviewing the verbatim interviews of 6 people with stroke, that were identified as representing the master plot overcoming the monster (the interview manuscripts were taken from research by Alwafi et al. [8]). Step 2 required authors to select vignettes that represented clear examples of the master plot. A.S developed the selection criteria for the vignettes identifying that the vignette could be selected if (a) verbatim statements from the participants interview script could clearly illustrate what the monster was and how it was overcome, (b) that the verbatim expression of the master plot could be easily arranged in a short paragraph, (c) the ‘monsters’ or experiences were considered by all authors as common for people with stroke. Alternatively, aspects of the master plot that were identified as striking, important or unusual. Finally, (d) each master plot selected had to be clearly distinguishable from the others chosen. Step 3. Once AS developed these plots based on the above criteria they were sent to other researchers for evaluation of clarity, and agreement. The three selected vignettes were labelled ‘Stories A, B, and C,‘ (Appendix A) but they represented the illness narratives that demonstrate 3 variations of the master plot overcoming the monster. The students were then requested to complete seven questions about each story (see Appendix B).

### Analysis

Descriptive statistical analysis were undertaken for Question 2 (Q2) including the number of times (n)each word was used and the percentage (%) of words occurrence in relation to the number of replies received. The open responses of the survey were analysed using a categorical-content narrative analysis [23]. This requires the researcher to breakdown open responses to self-contained areas of content. During content analysis it is normal to quantify the presence and meaning of certain words or themes e.g., [19]. The main researcher carried out the analysis (R.A). Author (AS) served as a ‘critical observer/ friend ' [24, 25], reflecting on and challenging explanations as they arose in respect to the data.

## Results

### Demographics of the respondents

A total of 90 students (39 MSc year 1; 32 BSc year 1; and 19 BSc final year 3 students) agreed to take part in this study. There were (25/90; 28%) male students with an average age of 25.0 years and (65/90, 72%) female students with a mean age of 20.1 years. Seventy-five (75/90, 83%) had not been on a neurological placement. Sixty students including all from BSc year 1 identified as having no placement experience. Table 1 provides a further break down of the group demographics.

**Table 1:** Demographics of the Respondents

Total 90	Frequency	percentage
**Gender**		
Male	25	27.8
Female	65	72.2
**Respondent’s Age**		
**17**	2	2.2
**18**	15	67.4
**19**	11	12.4
**20**	14	15.7
**21**	7	7.9
**22**	7	7.9
**23**	11	12.4
**24**	6	6.7
**25**	6	6.7
**26**	3	3.4
**28**	1	1.1
**29**	1	1.1
**30**	2	2.2
**31**	2	2.2
**34**	1	1.1
**No response**	1	1.1
**Year of Study**		
First year	71	78.9
Third year	19	21.1
**Course**		
MSc pre-	39	43.3
registration (year 1)	32	35.6
BSc (year 1)	19	21.1
BSc (year 3)		
**Neuro Placement**		
No	75	83.3
Yes	15	16.7
**Number of placements**		
Not Answered	1	1.1
No placement experiences	60	67.4

### Responses to each variation of the stories

A descriptive summary of student responses is given below and a summary of these responses is given within Table 2.

**Table 2:** A descriptive summary of student responses to individual questions

Story	Cohort	Year	Most attractive story (n & %)	Most unusual story (n & %)	Story the student would want to hear (n & %)	Story the student would want to tell (n & %)	Most common story (n&%)	Three most common words (frequency, % against number of students)		
A	BSc	1	6 (19%)	1 (3%)	14 (45%)	10 (32%)	20 (64%)	Motivational (19; 59%)	Family (9;29%)	Love (6; 19%)
	MSc	1	11 (28%)	2 (5%)	18 (46%)	14 (39%)	14 (36%)	Motivational (22; 56%)	Family (13;33%)	Sad (6;15.4%)
	BSc	3	1 (5%)	0	7 (36%)	8 (42%)	8 (42%)	Motivational (19; 59%)	Family (9;29%)	Love (6;19%)
B	BSc	1	14 (45%)	17 (55%)	1 (3%)	4 (13%)	6 (19%)	Sad (9;19%)	Despair (6;19%)	Neglected (6;19%)
	MSc	1	17 (44%)	13 (33%)	2 (5%)	5 (14%)	16 (41%)	Neglected (9; 23%)	Sad (8;20%)	mental health (7;18%)
	BSc	3	12 (63%)	5 (26%)	0	3 (16%)	4 (21%)	Despair (6;31%)	Negative [hospital experience] (4;21%)	Frustration (3;15%)
C	BSc	1	11 (35%)	12 (39%)	15 (48%)	14 (45%)	3 (10%)	Persistent (15;42%)	Motivational (6;19%)	Inspiring (6;19%)
	MSc	1	10 (25%)	22 (56%)	12 (30%)	12 (33%)	4 (10%)	Persistent (14; 36%)	Motivational (9;23%)	Positive (11;28%)
	BSc	3	6 (31%)	13 (68%)	12 (63%)	5 (26%)	2 (11%)	Persistent (8;42%)	Inspiring (6;31%)	Positive (6;31%)

Note: please note some % were affected by a missing number from the year 1 cohort: The most attractive, unusual want to hear and common columns had other answers and won’t add up to 100

The findings from narrative content analysis of the various stories along with the descriptive statistics are presented below.

#### Summary of Story A

the monster is identified through the challenges created by the Stroke and references mental health difficulties. The monster is overcome through motivation, emotional support and relationship with her daughter.

Story A appeared more attractive to the first-year students (MSc 28%, BSc 19%) compared to the final year BSc students where only one student (5%) identified this story as most attractive. The majority of students identified story A as the most common story of all three. Year 1 BSc students identified this as most often (20/31, 65%) with smaller amounts identified by other groups (year 3 BSc 8/19, 42%; year 1 MSc 14/39;36%). A similar number of first year BSc (14/31, 45%) and first year MSc students (18/39,46%) selected story A as the story they would prefer to hear. The BSc year 3 students selected it slightly less (7/19, 37%). Story A was most often selected as a preferred story to tell themselves if in a similar situation by final year BSc students (8/19, 42%) and MSc year 1 students (14/36, 39%). BSc year 1 students selected it less often (10/31, 32%). Many students would tell story A because they feel it would be the one that they would relate to the most. Only 3 students thought Story A was unusual. All three students were from the year 1 cohorts.

Most MSc students and BSc year 1 and 3 students felt uplifted and touched by the story and by the way the daughter supported her mother. This reaction was identified by around a half of year 1 BSc student (BSc 14/32; 50%) and slightly less prevalent for year 1 MSc students (12/39;31%) and in final year 3 BSc students (6/19;42%). For instance, one student stated, “*Story A. It is heartwarming, loving and touching*” (BSc Female Year 1 Student, P62). A majority of students across all student groups (BSc Year 3, 16/19; 84%; BSc Year 1, 22/32;69%; MSc Year 1, 29/39;74%) identified the importance and value of family members supporting the mental and physical well-being of the individual. Students often identified that the family member was a needed source of motivation to recovery and manage the impact of suffering two stroke. This was recognized by around two thirds of third year BSc (12/19,63%) but only in around a third of the other groups (MSc 12/39,31%; 1st year BSc 10/32,31%).

More year 1 students in both the BSc (11/32, 34%) and MSc (7/39, 17.9%) cohorts felt sad compared to the final year 3 students (2/19, 11%). However, the year 3 students identified as feeling empathy towards the individual (4/19, 21%) which was slightly similar to the year 1 MSc (6/39,15.4%,) and slightly more than the BSc year 1 students (4/32, 12.5%).

More final year BSc students (7/19, 37%) were able to identify how common it was for patients who had a stroke to lose motivation due to the length of recovery and frustrating nature of the process. This was less evident in the first-year groups (MSc 4/39,10%; BSc 2/32, 6%). Further to this, year BSc 3 students highlighted the considerable healthcare challenges following stroke (12/19, 63%) which was higher than the year one students (MSc 9/39, 23%; BSc 4/32, 13%). For instance, a final year student stated: “*The stroke was not fully treated and or she did not have the correct or personal recovery programme specifically for her case. The daughter family was the main rehab for her recovery not the untailored recovery [NHS] programme*”(MSc-preg Female Year 1, P20).

#### Summary of Story B

*The story identifies* d*ifficulties within the hospital experience as the monster; being able to recognize the difficulties and seeking to improve them was the way the monster was overcome.*

Story B was the story that was identified as most attractive across all students. The third-year BSc students appear to be the most drawn to this story (12/19, 63%), followed by the two first year cohorts BSc (14/31, 45%) and MSc students (17/39, 44%). A large number of MSc students selected story B as the most common story (16/39,41%), with the around 20% (BSc year 1 6/31, 19%; BSc year 3 4/19, 21%) of students identifying it as most common. Many students highlighted faults with the NHS that contributed to this being a common story. This included overwhelming stress on the NHS, the healthcare professional’s ability to implement screening protocols/procedures and negative culture in the wards. For instance, one student stated: “*Story B- I think there are a lot of people /patients that are seriously suffering in hospitals and finding it challenging to try and recover. The will to survive might be gone as well. That’s why it is essential to have a supportive environment to reignite their will to live*” (MSc male year 1 student, P24).

Students also recognised that it was common to hear unpleasant stories because stroke was regarded as a difficult experience to live through. A relatively low number of students selected Story B as the preferred story to tell (MSc year 1 5/36, 14%; BSc year 1 4/31, 13%; BSc year 3, 3/19, 16%). Story B was most often selected by students across the cohorts because it considered motivated people to be independent, persistent and self-driven to accomplish all their goals. One student stated: “*I would like to tell story B because this individual seems to have been extremely motivated by his stroke and they have achieved every goal set for them, which really inspires me and I hope by telling this story other people can be inspired too*” (BSc female year 1 student, P57). Another student stated: “*[story B] encourages people to do their own research and be persistent when asking doctors questions*” (MSc male year 1 student, P3).

This story considered was most unusual for the BSc first year students (17/31, 55%), followed by the MSc students (13/39, 33%) then the BSc year 3 students (5/19, 26%). The majority of year 3 BSc students highlighted the shortcomings of the hospital environment (14/19, 74%). Less numbers of year 1 students highlighted this although numbers were still considerable (MSc 24/39, 62%; BSc 14/32, 44%). The shortcomings identified by students including the healthcare professional’s misdiagnosis, inadequate care, patient neglect, a lack of communication between the patient and a lack of patient-centered care. The students were also critical of the ward environment on the mental health of the individuals; similar numbers highlighted this across programs (BSc year 3 6/19, 32%; BSc year 1 7/32 22%; MSc year 1 10/39 26%). One student illustrated this; “*[the story] highlights flaws in healthcare systems and shows how it affects patients and negatively impacts their recovery. Shows how patient centered care is sometimes not achieved and the needs and wishes of patients aren’t always taken into account*” (BSc female year 3 student, P72).

Students consistently identified this story with words that demonstrated their shock, annoyance and frustration at seeing the narrative reported by the individual. This was most prevalent for the year 1 BSc students (11/32, 34%) and less prevalent for the BSc year 3 students (5/19, 26.3%) and the year 1 MSc students (7/39, 19%). Interestingly, some third-year BSc students (4/19, 21%) identified the story as realistic and were able to identify the mistakes as unfortunate but understood it from the perspective of broader challenges with the NHS and problems faced like short staffing and patient overload. One student stated; “*The patient shed light to the unfortunate but realistic experiences that happens to stroke patients where their stroke might have been missed in the early stages*” (BSc male year 3 student, P80). Students identified that they felt empathy towards the individuals this was most often in BSc cohorts (year 1 11/32, 34%; year 3 10/19, 53%) and less often in the MSc cohort (7/39, 19%).

***Summary of Story C.****The monster was identified as a loss of control following the stroke and the identification of negative or challenging experiences e.g., the inability to speak or express herself. A central way the monster was overcome was to view negative experiences as an adventure. Life and new experiences become an adventure*.

Students were equally attracted to story C (BSc year 1 11/31, 35%; BSc year 3 6/19, 32%; MSc year 1 10/32, 25%). Around 10% of all students identified story C as the most common (MSc year 1 4/39, 10%; BSc year 1 3/31, 10%; BSc year 3 2/19, 11%). Those that identified the story as common did so by referencing the common physical and cognitive problems that many stroke patients experience such as losing ability to perform basic activities, speech, reduced memory and concentration. Story C was also identified as common due to the ability of people to adjust to one’s viewpoint and mindset after a stroke. The BSc year 3 students most often selected story C as their preferred story (12/19, 63%). This was similar to the year 1 BSc students who most often selected it (15/31, 48%). Slightly less students from the MSc cohort identified C as the preferred story (12/39, 31%). Students valued particular characteristics demonstrated in this story including persistence and determination to succeed. Also, students said that motivation would reassure respondents that their loved ones would be determined to progress and persist with their rehabilitation programme in order to achieve the best quality of life possible. BSc year 1 students stated that they would tell Story C if they were in a similar situation (14/31, 45.2%). This was slightly less from other cohorts (MSc year 1 12/36, 33%; BSc year 3 5/19, 26%). The value of the story for these students was the perceived ability to demonstrate the capacity to evolve and cope with a life-changing disease in a positive and novel way by viewing life as an adventure. For instance, one student stated: “… *because it is a story of hope and is encouraging that things will end up good*” (MSc-preg Female Year 1 Student, P14). Despite it being preferred, the majority of students across cohorts identified Story C as the most unusual for two cohorts (BSc year 3 13/19, 68%; MSc year 1 22/39, 56%), and perceived as unusual by less BSc year 1 students (12/31, 39%). Story C was assumed to be unusual due to the individual’s positive and hopeful outlook on life and seeing how life and recovery journey was an excitement and adventure.

High numbers of all year groups (year 1 BSc 18/32, 56%; year 1 MSc 16/38, 42%, n = 1 missing data; year 3 BSc 14/19, 74%) perceived that the story illustrated an amazingly rare positive attitude and mindset following the stroke. Several students found the story motivating and inspiring to read (BSc year 3 7/19, 37%; BSc year 1 7/32, 22%; MSc year 1 4/38, 11%). Interestingly this was identified as a surprising story for a number of final year students as it was unexpected and represented a unique ideology (8/19, 42%) whereas only one student in the year 1 cohorts identified it as surprising. At least half or more of all students identified the characteristics of the individual of this story most often. They highlighted the ability of someone to find strength, determination, and motivation intrinsically, enabling her to persevere with the rehabilitation program to move forwards in her recovery journey from stroke. This was illustrated by similar response levels across groups (BSc year 3, 12/19, 63%; MSc year 1 19/38, 50%; BSc Year 1 15/32, 47%).

## Discussion

To the best of the authors knowledge this is the first study to consider how physiotherapy students respond to the master plot ‘overcoming the monster’. Physiotherapy students consistently wanted to hear story A it and could identify with it and with the importance of family. Final year students identified empathy with story A, whilst more first year students identified feeling sad because of this story. A large proportion of students were attracted to story B, but often did not want to tell it. A moderate proportion of students across cohorts were attracted to story C and valued the characteristics of the person who told them. However, many final year students were surprised that it would be told.

Physiotherapy students recognized the challenges and poor experience of inpatient care identified in Story B. Third year and pre-reg MSc students appeared to be aware of the challenges facing the National Health Service (NHS) that included lack of staffing across all health care professionals, an excessive workload, an inadequate level of resources, and training, all of which have an impact on building a therapeutic relationship and the ability to provide high-quality patient care [26]. This may suggest clinical exposure and age may influence the expectations of healthcare experience, something that has been identified in past research [27]. For example, younger newly qualified nurses evaluated their work environment more positively, whereas senior registered nurses evaluated their workplace more negatively [27].

There were several important comparisons that this study has with past research on students’ responses to narrative types. In the current study, physiotherapy students did not question whether the patient accepted what had happened or if the story was realistic. This is in contrast to past research. For instance, in another vignette study by Soundy [19] physiotherapy students viewed a ‘quest master plot’ and questioned if acceptance had occurred and considered a ‘restitution master plot’ and questioned if it was actually realistic. Further students who heard the ‘chaos narrative’ identified the individual as depressed. It is important to understand that, in the current study, students did not use such ‘labels’ which can infer a judgement to the nature of the master plot or the person telling it. Rather, physiotherapy students appeared empathetic to the plot.

Health care professionals can influence which narrative types exist within an inpatient or outpatient environment [28]. Interactions with patients should provide opportunity to consider peoples’ stories and health care professionals should be aware of their own preference for particular master plots. This awareness could help isolate any bias towards particular narratives when listening to a patients’ narrative [19, 21]. It is also important for students and health care professionals to understand how a patient defines the plot may be different from how they define it. For instance, an individual in the study by France et al. [21] regarded himself as ‘cured’ despite an ongoing disability. The current findings support the importance of sharing stories rather than seeking to correct stories [15].

Empathy was consistently expressed towards story A and story B. Reference in story A to a family member and reference to poor experiences with the hospital environment in Story B appeared to be aspects which physiotherapy students could empathise with. This is supported by past research [29]. Empathy is one of the core psychosocial skills that can improve patients’ psychosocial outcomes, including adherence and patient confidence [30]. It is important to understand what master plots generate empathy and what do not. For instance, a reduction in empathy has been previously associated with the chaos narrative by physiotherapy students [19]. This is important since this story is associated with the severity of cognitive impairment or the want of patients to overtly consider their own experiences following a stroke [31]. However, stories that are inspiring and reveal a positive recovery message are met with a positive response by health care students [32]. Overcoming the monster master plot looks at the past to identify how someone overcame a difficulty. The plot reveals the persons character in a positive light and also reveals their past struggles. The master plot does not challenge rehabilitation goals that had been set, during a student-patient interaction. Contrastingly, stories like the chaos master plot are ‘lodged’ in the present, or stories like the restitution and quest look to the future and could be perceived as impacting rehabilitation goals and the student-clinician interaction [19]. Further it is important to recognise that students’ reactions can also be from inherent biases [9]. For instance, vignettes with a patient educational level, culture and ethnicity can generate different responses from students [33]. Hence training using narratives should encourage less judgement of challenges faced by patients and more understanding of how challenges are presented and the range of stories that can be told [34]. Being able to understand that the patients are more than their disease can help students listen more and positively impact patient care [35, 36].

### Implications

Physiotherapy students appeared to be empathic to understanding the ‘monster’ or challenges faced by patients who have had a stroke. Giving time within interactions to do this will help build therapeutic relationships. Physiotherapy students need to create environments where stories can be told, and the personal challenges of patients understood. Physiotherapy students could benefit from time to self-reflect on their responses to different narrative master plots during clinical placements.

There may be master plots told by patients that evoke a response from the student physiotherapists where the student perceives a need to correct the story (using positive interventions). However, student physiotherapists should consider that the telling of the story is therapeutic, it builds trust and the individual’s story will likely evolve with time and opportunities to share and listen to other stories. Training programmes need to include training which highlights how implicit bias and bias towards particular master plots may influence the students’ reaction.

Clinical placement education that offers opportunities to listen and understand experiences of stroke would be of great value to student physiotherapists. Patients may need time and space as well as direct questions within interactions to identify a monster and the understanding that the ability to overcome the monster may evolve over time.

### Limitations

Individuals presenting this narrative were identified from a group who had a long time to recover and less challenges to narrate their story. How common ‘overcoming the monster master plot’ is needs further consideration. The current manuscript is focused on one plot and how other master plots evolve or how they interact with this master plot is not established. Responses to each narrative appeared to vary with clinical experience and potentially age. Further research is needed to develop understanding of both these factors. Numbers across groups were not equal and different methods were used for completing the survey. This may limit a fuller understanding of the responses by the final year students. The content analysis focused on the most common expressions by student. This process may have limited the focus of the current results. The generalisability of this work to other healthcare students has not been established due to the specific population of students included. Finally, the effects of aphasia and inability to narrate due to a stroke is not considered within the current manuscript.

## Conclusion

Students responded to the master plot of overcoming the monster with more empathy compared to other narrative master plots. This is significant because it emphasizes how crucial it is for students to understand the patients’ stories and difficulties or ‘monsters’ encountered. It is important that physiotherapy students take time to identify what the ‘monster’ may be for a patient, and this requires time for getting to know a patient and their story. It appears more important to encourage students to listen and understand challenges, to identify the problems in the story that may not fit currently with the goals of stroke rehabilitation.

## Electronic supplementary material

Below is the link to the electronic supplementary material.

Supplementary Material 1

## Data Availability

Data is available in the supplementary file.

## Declarations

Nothing to declare.
