# Supplementary material for: Overcoming the monster! Perceptions of physiotherapy students regarding the use of stroke master plots for building therapeutic relationships; a vignette study
Source: BMC Med Educ. 2023 May 5;23:311. doi: 10.1186/s12909-023-04301-5 (PMC10161626; doi:10.1186/s12909-023-04301-5)
Supplement: Supplementary file 1 — Supplementary Material 1 [file 12909_2023_4301_MOESM1_ESM.docx]

**Appendix A.**

**The Narrative Vignette**

Please read the following passage which is a real-life account of an individual who had suffered a stroke. The individual is describing a particular story plot from their journey following rehabilitation, the story identifies the key challenge they face. Please take a moment to consider the story and then for each story please answer the questions below.

***Story (A)***

This experience was identified by an individual who had two strokes. The first was 4 years ago.

*“I then [following discharge from the Stroke ward] returned home with medication and a low salt diet. I started to working to get low blood pressure and scheduled a visit with my doctor. But then I still continued to get complications from the other stroke… I thought I was on the journey to recovery… and it took an awful long time to come around and understand what happened…I was, obviously, frustrated and depressed and disheartened as well. I needed motivation to carry on…I’ve got a daughter, she’s a real inspiration for me. She kept me going and she just brought some joy, which has really enabled me to carry on. I think that’s what did it for me, because I think she understood what I was going through. And she became really helpful and encouraging to keep me motivated. And I'm glad I had my daughter to…encourage me to carry on to this day. And that’s why I feel I'm a survivor, a real survivor. I could sense that she wanted me to carry on. Because it’s that relationship, mother and daughter relationship. She didn’t want to lose me, I didn’t want to lose her, really, because we’re very closely bonded together*.”

***Story (B)***

This experience was identified by an individual who had an ischemic stroke 15 years ago.

“*They investigated me for an upper spinal tract injury because I’d had an RTA [road traffic accident]. They didn’t really look at what was in front of them at all, which was classic Stroke symptoms. I was put on an observation ward for 72 hours and nothing else was done. And then after three days the on-duty registrar ordered an MRI and that’s when the stroke was discovered*…. *But those mistakes are still being made today. And that’s what motivates me to keep getting involved in as much as I can*….*I mean hospital; I was on a ward with octogenarian, double incontinent men. It wasn’t the most pleasant of experiences. I think it probably, it didn’t destroy me, I was on the edge of that. I had told a number of clinical academic colleagues that I’ve made in the last 15 years, I can remember at one point waking up and this awful stench of urine and faeces just permeating every orifice of my body. And pulling the bedclothes over my head and praying that I’d go to sleep and not wake up. Now, that’s not me at all, but that was the effect of that environment. And when I was told by my consultant that they’d actually discussed in a meeting about my psychological welfare being on that ward, and decided that I was coping okay. But nobody had asked me…I still think that we have to acknowledge that we must give hope. I don’t accept this thing, you may be giving false hope and I answer that now as well, no one talks about false despair. And I think false despair is the evil twin of false hope. By not giving hope we’re compelling people into this vortex of false despair, which is far more destructive*…“*I’ve achieved every goal that I’ve set myself, other than doing the physical cycle ride to Paris, which I realised was a non-starter in 2014. So I sort of gave up on that particular one. They started in hospital as the most basic, primitive drives. Being able to take myself to the lavatory by myself was my first goal. I was fed-up of people I didn’t know having to support me in that very, very basic human function*.

***Story (C)***

This experience was identified by an individual who had an ischemic stroke 16 years ago.

“*when I was in hospital I didn’t know much what was happening in the first month. I couldn’t do anything. That was awful. It’s so humiliating in fact because you’re slightly aware but not completely. But afterwards the difficulty I had was with my voice. I couldn’t – I didn’t make any sound at first, in fact for about a month and a half I had no sound coming out. But I thought I was speaking. And then it got better but it’s very, it’s still, as you can hear, very bad…I read, a man who was, he was some kind of trainer I suppose in the SAS talking about the types of people that they liked. And one of the things he had said was determination to do, to achieve was not enough. It was persistence. Then I have discovered that with persistence – if I don’t do my exercises regularly I go backwards. So that has really helped me that I have to be always persistent…Because if you think of young children, when they like, I remember my children when they were trying to walk they just persisted and persisted. And they didn’t mind falling over because I suppose we all said, “Oh, well done,” and what have you…*“*I’m still coming to terms with it…. But I don’t mind that. I like, it’s kind of an adventure to me*…. *Well, I have had the advantage of falling over – this is four years before my stroke. I fell over and I smashed my face. I tripped on a paving stone. And I lost my sight totally in the eye….And from that time on it gave me the advantage of looking at life as learning new things. And in fact I almost wouldn’t say, apart from I want to read desperately, apart from that, I have had lots of things that I call adventures. Meeting people unexpectedly in the street and being taken for coffee and finding out about them has been really fascinating actually…So it programmed me for when I came out of hospital I looked at everything as excitement and people are curious about you when you can’t, when you're fuddling around really.*”

**Appendix B: The vignette questionnaire**

***Instructions for questions***

Please answer the following questions and feel free to re-read any parts of the above stories again. There are no right or wrong answers to the following questions as it is your opinions that matter. Therefore, do not feel your replies must fill the spaces provided, or if necessary, please continue your answer on the other side of the paper if you do not have enough space.

**Your Identification number** (so we can locate your answer an remove if needed)

Please identify a unique number for yourself and retain it: identify a memorable name or word and add this number to the current date with an underscore e.g., buffalo (YOU detail this)_16/5/2021. Send these details to Dr X, the lead investigator.

Identification number:_______________

Before undertaking this survey please confirm (by clicking a tick box) you have read the accompanying information sheet and has opportunity to ask any questions you may want answers to and that you consent:

Tick box

**Please indicate your gender (please delete)**

- Male
- Female

**How old are you?  (Years)**

**Year of the studying (please delete one)**

- First year
- Final year

Have you undertaken a neurological placement?

Yes / No

How many placements have you undertaken?

Number:

*Questions applicable to Stories A, B and C*

(1) What is your immediate reaction to each story?

Story(**A)**

Story **(B)**

Story **(C)**

(2) list three or more words that describe each story.

| Story | Words |
| --- | --- |
| Story A |  |
| Story B |  |
| Story C |  |

*Other Questions*

(3) What story attracts your attention the most and why?

(4) Which story do you think is the most common and why?

(5) Which story do you think is the most unusual and why?

(6) If the stroke incident described above happened to a close relative or friend, which of the three stories (A, B, or C) would you prefer to hear them tell and why?

(7) If you experienced this life-changing disease, which story do you think you would tell and why?
